# Types of Physical Activity Recommended by Primary Care Providers for Patients at Risk for Cardiovascular Disease

**DOI:** 10.5888/pcd18.200545

**Published:** 2021-05-06

**Authors:** John D. Omura, Kathleen B. Watson, Fleetwood Loustalot, Janet E. Fulton, Susan A. Carlson

**Affiliations:** 1 Division of Nutrition, Physical Activity, and Obesity, National Center for Chronic Disease Prevention and Health Promotion, Centers for Disease Control and Prevention, Atlanta, Georgia; 2 Division for Heart Disease and Stroke Prevention, National Center for Chronic Disease Prevention and Health Promotion, Centers for Disease Control and Prevention, Atlanta, Georgia

## Abstract

Primary care providers (PCPs) are uniquely positioned to promote physical activity for cardiovascular health. We sought to determine the types of physical activity that PCPs most often recommend to patients at risk for cardiovascular disease (CVD) and how these recommendations vary by PCPs’ physical activity counseling practices. We examined the types of physical activity (walking, supervised exercise sessions, or other) PCPs most often suggested for CVD prevention among respondents to the 2018 DocStyles survey (N = 1,088). Most PCPs (80.0%) suggested walking to their patients at risk for CVD; however, PCPs who infrequently discussed physical activity with their patients at risk for CVD suggested walking less often than those who more frequently discussed physical activity. Walking is an easy and low-cost form of physical activity, and opportunities exist for certain PCPs to promote walking as part of their physical activity counseling practices for CVD prevention.

**SUMMARY**

**What is already known on this topic?**

Walking presents an important opportunity for cardiovascular disease (CVD) prevention, and primary care providers (PCPs) are uniquely positioned to promote walking.

**What is added by this report?**

Most PCPs suggest walking for physical activity for their patients at risk for CVD. However, this varies by the PCP’s sex, age, years in practice, and the socioeconomic status of their patients.

**What are the implications for public health practice?**

Important opportunities exist for PCPs to promote walking as part of physical activity counseling for CVD prevention.

## Objective

Physical inactivity is an important modifiable risk factor for cardiovascular disease (CVD), and health care providers can contribute to CVD prevention by providing intensive behavioral counseling to at-risk patients that includes promoting physical activity (1). Yet only 59% of primary care providers (PCPs) discuss physical activity with their at-risk patients (2).

Although behavioral counseling recommendations do not specify types of physical activity, walking is a simple activity to suggest because it is an easy way for most adults to be active (1,3,4). Understanding the types of physical activity PCPs suggest to patients at risk for CVD by PCP characteristics and their physical activity counseling practices can identify opportunities for certain PCPs to promote physical activity through walking. Therefore, we sought to determine the types of physical activity PCPs most often suggest to their patients at risk for CVD and examine variation by physical activity counseling practices.

## Methods

DocStyles is a web-based panel survey conducted by Porter Novelli Public Services; we used data from the June to August 2018 survey (5). Panelists from Sermo’s Global Medical Panel (www.sermo.com) were invited to take the 2018 survey on the basis of their past survey participation level. High responders — those who completed more than 75% of surveys they were sent — were invited first, followed by those who answered 25% to 75% of surveys and those who answered fewer than 25%. Panelists who did not participate in the previous year’s DocStyles survey were prioritized above the previous year’s respondents to help facilitate broad coverage among panelists. Respondents were paid an honorarium, which varied from $40 to $90 on the basis of the number of questions asked. Respondents could exit the survey at any time.

Quotas were set for provider types (ie, primary care physicians, nurse practitioners). A total of 3,465 health care providers were invited to participate, and 2,256 completed the entire survey. We included only primary care physicians and nurse practitioners (n = 1,253) because our focus was on physical activity counseling for patients at risk for CVD. We excluded respondents who reported mainly working in an inpatient practice (n = 161) and those who never discussed physical activity (n = 4), resulting in a final sample of 1,088.

To assess the percentage of patients with CVD with whom PCPs discussed physical activity, participants were asked, “With how many of your at-risk patients do you discuss physical activity?” Response options were “none” (n = 4), “few (1%–25%)” (n = 51), “some (26%–50%)” (n = 138), “many (51%–75%)” (n = 237), or “most (>75%)” (n = 662). Our analysis excluded respondents who selected none.

The survey asked PCPs to select the type of physical activity they most often suggested to patients at risk for CVD. Respondents were grouped into 1 of 3 activity categories on the basis of their selection: walking, supervised sessions, or other. 

We examined the prevalence of suggesting each type of physical activity overall, and by PCP characteristics and the percentage of patients at risk for CVD with whom they discussed physical activity. Pairwise *t* tests and orthogonal polynomial contrasts identified significant differences and trends in the prevalence of suggesting physical activity types. Values of *P *< .05 were considered significant. Analyses were conducted by using SUDAAN, Release 11 (RTI).

## Results

Overall, 80.0% of PCPs most often suggested walking to their patients at risk for CVD, 7.9% suggested supervised exercise sessions, and 12.1% suggested another type of activity (Table). Prevalence of the type of activity suggested varied by PCP age group, sex, race/ethnicity, years in practice, and financial status of most patients in their practice. The types of activities PCPs most often suggested also differed by the percentage of patients at risk for CVD with whom they discussed physical activity (Figure). Prevalence of suggesting walking increased in a nonlinear trend as the percentage of patients increased. However, prevalence of suggesting supervised exercise sessions or other types of activity decreased linearly as this percentage increased.

**Table:** Types of Physical Activity PCPs Most Often Recommended to Patients at Risk for CVD, by PCP Characteristics, DocStyles 2018^a^

PCP Characteristic	Sample Size	Type of Physical Activity, % (95% CI)		
		Walking	Supervised Sessions	Other
**Total**	1,088	80.0 (77.5–82.2)	7.9 (6.4–9.7)	12.1 (10.3–14.2)
**Age, y^b^**				
<45	385	72.7 (68.1–76.9)	10.6 (7.9–14.2)	16.6 (13.2–20.7)
≥45	703	83.9 (81.0–86.5)	6.4 (4.8–8.5)	9.7 (7.7–12.1)
**Sex^c^**				
Men	574	77.0 (73.4–80.3)	9.4 (7.3–12.1)	13.6 (11.0–16.6)
Women	514	83.3 (79.8–86.3)	6.2 (4.4–8.7)	10.5 (8.1–13.5)
**Race/ethnicity^d^**				
Non-Hispanic White	743	82.0 (79.0–84.6)	6.7 (5.1–8.8)	11.3 (9.2–13.8)
Other	345	75.7 (70.8–79.9)	10.4 (7.6–14.1)	13.9 (10.6–18.0)
**Region^e^**				
Midwest	372	82.0 (77.7–85.6)	7.3 (5.0–10.4)	10.8 (8.0–14.3)
South	251	77.3 (71.7–82.1)	8.8 (5.8–13.0)	13.9 (10.2–18.8)
Northeast	255	78.4 (73.0–83.1)	8.2 (5.4–12.3)	13.3 (9.7–18.1)
West	210	81.4 (75.6–86.1)	7.6 (4.7–12.1)	11.0 (7.4–16.0)
**Specialty**				
Family practice	470	80.0 (75.8–83.7)	8.1 (5.8–11.2)	11.9 (9.1–15.5)
Internal medicine	395	78.7 (74.8–82.2)	8.7 (6.5–11.6)	12.6 (9.8–15.9)
Nurse practitioner	223	82.5 (76.9–87.0)	5.8 (3.4– 9.8)	11.7 (8.1–16.6)
**Years in practice^f^**				
3–10	285	72.6 (67.2–77.5)	9.8 (6.9–13.9)	17.5 (13.5–22.4)
11–20	378	79.9 (75.5–83.6)	10.1 (7.4–13.5)	10.1 (7.4–13.5)
>20	425	84.9 (81.2–88.0)	4.7 (3.1–7.2)	10.4 (7.8–13.6)
**Main practice setting**				
Individual practice	843	80.8 (78.0–83.3)	7.6 (6.0–9.6)	11.6 (9.6–14.0)
Group practice	245	77.1 (71.5–82.0)	9.0 (6.0–13.3)	13.9 (10.1–18.8)
**Privileges at teaching hospital**				
Yes	423	78.3 (74.1–81.9)	8.0 (5.8–11.0)	13.7 (10.7–17.3)
No	665	81.1 (77.9–83.9)	7.8 (6.0–10.1)	11.1 (8.9–13.8)
**Financial status of majority of patients^g^**				
Poor to low middle class	350	81.7 (77.3–85.4)	5.4 (3.5–8.4)	12.9 (9.7–16.8)
Middle class	440	81.8 (77.9–85.2)	8.2 (6.0–11.1)	10.0 (7.5–13.2)
Upper middle class to affluent	298	75.2 (69.9–79.8)	10.4 (7.4–14.4)	14.4 (10.9–18.9)

Abbreviations: CVD, cardiovascular disease; PCP, primary care provider.

a DocStyles (5). The survey defined patients at risk for CVD as patients who 1) are overweight or have obesity and 2) have hypertension, dyslipidemia, impaired fasting glucose, or the metabolic syndrome. PCPs were asked, “What type of physical activity do you most often suggest to your at-risk patients?” Respondents who selected either gym sessions with an exercise professional or group exercise classes were categorized as selecting supervised sessions. Respondents who selected swimming, yoga/tai chi, bicycling, or other/none of these were categorized as other.

b Significant pairwise differences (all *P* < .05): walking (<45 vs ≥45), supervised sessions (<45 vs ≥45), and other (<45 vs ≥45).

c Significant pairwise differences (all *P* < .05): walking (men vs women) and supervised sessions (men vs women).

d Significant pairwise differences (all *P* < .05): walking (non-Hispanic White vs other) and supervised sessions (non-Hispanic White vs other).

e Region was based on the state in which PCPs reported they lived. Midwest: Illinois, Indiana, Iowa, Kansas, Michigan, Minnesota, Missouri, Nebraska, North Dakota, Ohio, South Dakota, and Wisconsin; Northeast: Connecticut, Maine, Massachusetts, New Hampshire, New Jersey, New York, Pennsylvania, Rhode Island, and Vermont; South: Alabama, Arkansas, Delaware, District of Columbia, Florida, Georgia, Kentucky, Louisiana, Maryland, Mississippi, North Carolina, Oklahoma, South Carolina, Tennessee, Texas, Virginia, and West Virginia; West: Alaska, Arizona, California, Colorado, Hawaii, Idaho, Montana, Nevada, New Mexico, Oregon, Utah, Washington, and Wyoming.

f Significant pairwise differences (all *P* < .05): walking (3–10 vs 11–20; 3–10 vs >20), supervised sessions (3–10 vs >20), and other (3–10 vs 11–20; 3–10 vs >20).

g Respondents were asked to select the category that best described the approximate financial situation (annual household income) of most of their patients. Response options included poor (less than $25,000), lower middle ($25,000–$49,999), middle ($50,000–$99,999), upper middle ($100,000–$249,999), or affluent (≥$250,000). Respondents who selected poor or lower middle were grouped into the “poor to low middle class” category; those who selected middle were grouped into the “middle class” category; and those who selected upper middle or affluent were grouped into the “upper middle class to affluent” category. Significant pairwise differences (all *P* < .05): walking (poor to low middle class vs upper middle class to affluent; middle class vs upper middle class to affluent) and supervised sessions (poor to low middle class vs upper middle class to affluent).

**Figure:**
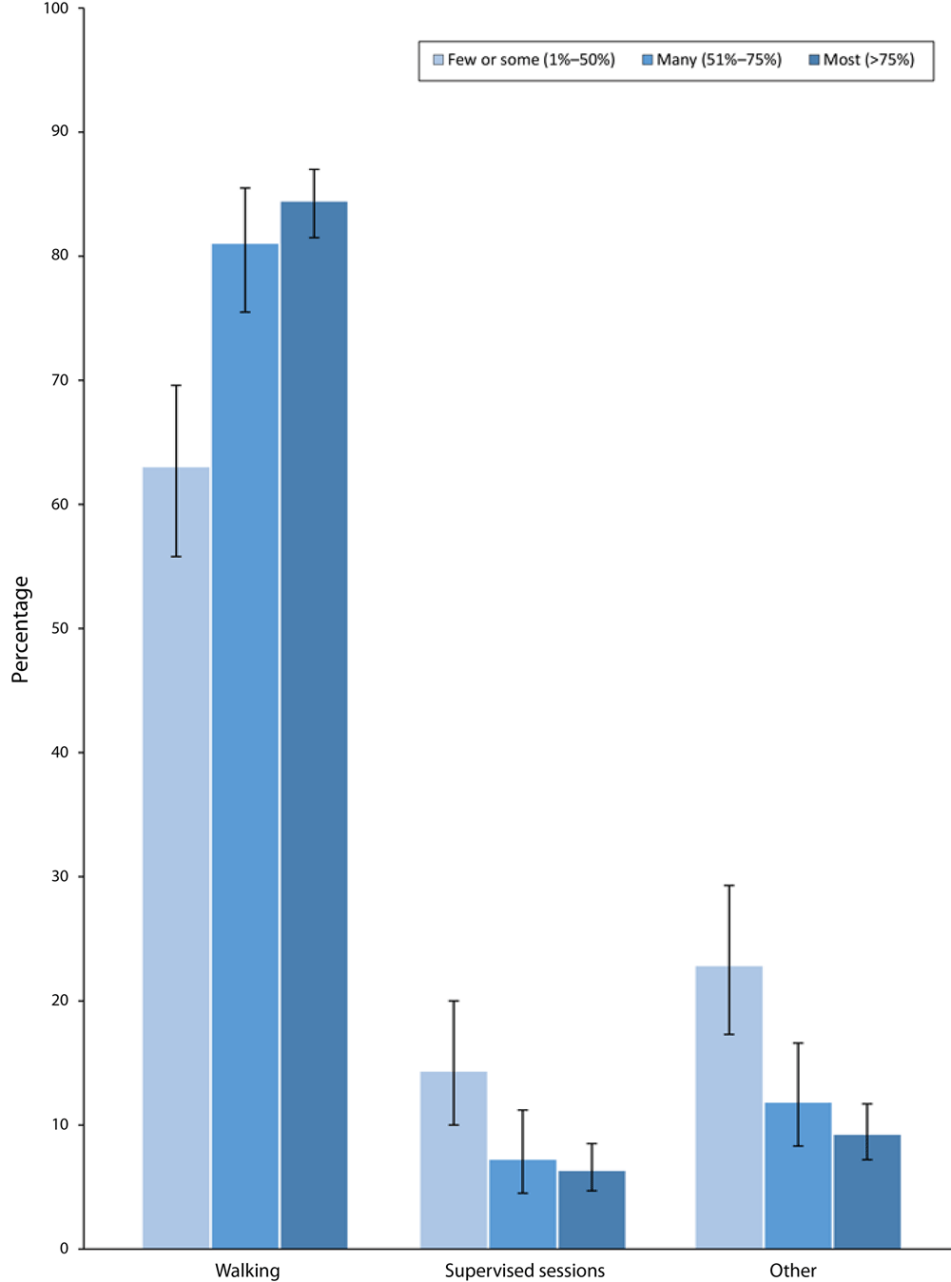
Prevalence of types of physical activity primary care providers (N = 1,088) most often suggested to patients at risk for cardiovascular disease (CVD), by percentage of patients with whom they discussed physical activity. Patients at increased risk for CVD were defined as those who were overweight or had obesity, hypertension, dyslipidemia, impaired fasting glucose, or the metabolic syndrome. Linear and quadratic trends for walking were significant at *P* < .05. Linear trend was significant for supervised exercise sessions and other exercise at *P* < .05. Error bars indicate 95% CIs. Source: DocStyles 2018 (5).

**Table:** 

Patients With Whom Primary Care Providers Discussed Physical Activity, %	Walking,% (95% CI)	Supervised Sessions, % (95% CI)	Other Exercise, % (95% CI)
>75	84.4 (81.5–87.0)	6.3 (4.7–8.5)	9.2 (7.2–11.7)
51–75	81.0 (75.5–85.5)	7.2 (4.5–11.2)	11.8 (8.3–16.6)
1–50	63.0 (55.8–69.6)	14.3 (10.0–20.0)	22.8 (17.3–29.3)

## Discussion

Our findings highlight opportunities for certain PCPs to suggest walking as an easy form of activity for CVD prevention as part of their counseling. Health care professionals can contribute to CVD prevention by counseling their patients about physical activity (1); however, only 59% of PCPs in our study discussed physical activity with most of their at-risk patients, which is consistent with previous studies (2). Promoting walking may affect public health because adults at high CVD risk are known to participate in low levels of physical activity, and walking is an easy way for most adults to be physically active (3,6–8). We observed that PCPs who counseled a smaller proportion of their patients at risk for CVD about physical activity less frequently suggested walking, while the opposite was observed for supervised sessions. This suggests that PCPs who do not regularly provide physical activity counseling to their patients at risk for CVD may tend to recommend more involved forms of physical activity. Walking can offer these PCPs a broadly available and accessible form of physical activity to include in their behavioral counseling (3).

We observed several differences in the types of physical activity recommended by characteristics of PCPs and their practices. For example, PCPs who were women and aged 45 or older were more likely to suggest walking than those who were men and younger, which is similar to walking practices among the general population (9,10). These findings suggest that PCPs who might walk more themselves may be more likely to suggest walking to patients, consistent with research showing that health care providers who are physically active are more likely to provide physical activity counseling (11). In addition, we observed that PCPs with most patients of high socioeconomic status more commonly suggested supervised exercise sessions than PCPs primarily serving patients of low socioeconomic status, suggesting that PCPs may tend to recommend supervised sessions to those who they perceive can afford them. Walking is a simple and low-cost form of physical activity to recommend (3).

A strength of our study was that DocStyles is a large, nationwide survey allowing for comparison by provider characteristics. A limitation was that the sample was not nationally representative; therefore, results may not be generalizable. However, our analytic sample of physicians was similar to the 2018 DocStyles sample and the American Medical Association master file (5). 

Walking is an easy and low-cost form of physical activity that PCPs can suggest to their patients at risk for CVD. Although most PCPs do suggest walking, important differences exist by provider characteristics. Our study highlights opportunities for certain PCPs to promote walking as part of their physical activity counseling practices for CVD prevention.
